# Human T-cell leukemia virus type-1-encoded protein HBZ represses p53 function by inhibiting the acetyltransferase activity of p300/CBP and HBO1

**DOI:** 10.18632/oncotarget.6424

**Published:** 2015-11-28

**Authors:** Diana G. Wright, Claire Marchal, Kimson Hoang, John A. Ankney, Stephanie T. Nguyen, Amanda W. Rushing, Nicholas Polakowski, Benoit Miotto, Isabelle Lemasson

**Affiliations:** ^1^ Brody School of Medicine, Department of Microbiology and Immunology, East Carolina University, Greenville, NC, USA; ^2^ Université Paris Diderot, Sorbonne Paris Cité, Epigenetics and Cell Fate, UMR 7216, CNRS, Paris, France; ^3^ Department of Biochemistry and Biophysics, University of North Carolina at Chapel Hill, Chapel Hill, NC, USA; ^4^ INSERM, U1016, Institut Cochin, Paris, France; ^5^ CNRS, UMR8104, Paris, France; ^6^ Université Paris Descartes, Sorbonne Paris Cité, Paris, France

**Keywords:** human T-cell leukemia virus type-1, adult T-cell leukemia, HBZ, histone acetyl transferase activity, p53

## Abstract

Adult T-cell leukemia (ATL) is an often fatal malignancy caused by infection with the complex retrovirus, human T-cell Leukemia Virus, type 1 (HTLV-1). In ATL patient samples, the tumor suppressor, p53, is infrequently mutated; however, it has been shown to be inactivated by the viral protein, Tax. Here, we show that another HTLV-1 protein, HBZ, represses p53 activity. In HCT116 p53^+/+^ cells treated with the DNA-damaging agent, etoposide, HBZ reduced p53-mediated activation of p21/CDKN1A and GADD45A expression, which was associated with a delay in G2 phase-arrest. These effects were attributed to direct inhibition of the histone acetyltransferase (HAT) activity of p300/CBP by HBZ, causing a reduction in p53 acetylation, which has be linked to decreased p53 activity. In addition, HBZ bound to, and inhibited the HAT activity of HBO1. Although HBO1 did not acetylate p53, it acted as a coactivator for p53 at the p21/CDKN1A promoter. Therefore, through interactions with two separate HAT proteins, HBZ impairs the ability of p53 to activate transcription. This mechanism may explain how p53 activity is restricted in ATL cells that do not express Tax due to modifications of the HTLV-1 provirus, which accounts for a majority of patient samples.

## INTRODUCTION

The tumor suppressor, p53, is a central regulator of genome stability in mammalian cells. Following DNA damage, p53 becomes acetylated and phosphorylated at multiple sites, thereby shifting the protein from an unstable, latent form to one that is stable, active and concentrated in the nucleus [1]. This transition allows p53 to bind to promoters of genes involved in cell cycle arrest, such as p21/CDKN1A and GADD45A, and activate their transcription [2–4]. p53 is also able to regulate expression of genes involved in the DNA damage response, apoptosis and in its own regulation [5, 6]. Although certain posttranslational modifications may serve redundant functions, there appears to be separate patterns of acetylation and phosphorylation of p53 that favor either cell cycle arrest and DNA repair or, alternatively, apoptosis [6]. Regardless of these complexities, it is clear that acetylation of p53 is essential in promoting either of the two cellular fates [7].

In general, acetylation has been shown to augment the transcriptional activity and stability of p53. For example, acetylation of lysine residues within the C-terminal domain of the protein have been found to induce conformational changes that may enhance DNA-binding activity and interactions with other transcriptional regulatory proteins [8–10]. In addition, acetylated lysine residues in p53 may be shielded from ubiquitination by Mdm2 and other E3 ubiquitin ligases that mark p53 for proteosomal degradation [11, 12]. Furthermore, these modified lysine residues may enhance interactions between p53 and coactivators, thereby facilitating the recruitment of these coactivators to promoters [13, 14].

p300 (also designated KAT3B), was the first histone acetyltransferase (HAT)-carrying protein found to acetylate p53 [9]. Of the six HAT proteins reported to acetylate p53, p300 is responsible for the most extensive acetylation of this factor, modifying five lysine residues in the C-terminal domain of p53 (K370, K372, K373, K381 and K382) [9], one lysine residue in the DNA-binding domain (K164) [7], and one lysine residue positioned between these two domains (K305) [15]. This observation implies an important role for p300 in regulating p53 activity, as acetylation of different lysine residues in p53 may elicit redundant effects [10], and at least partial acetylation of p53 is required for its transcriptional activity. Acetylation of p53 by p300 frequently contributes to activation of p21/CDKN1A expression, promoting cell cycle arrest, while effects on apoptotic gene expression, such as that of PUMA, appear to vary with cell type [16]. In addition to directly modifying p53, p300 contributes to p53-mediated transcriptional activation *via* its coactivator function and through histone acetylation at promoters bound by p53.

Another HAT-containing protein, histone acetyltransferase bound to ORC1 (HBO1, KAT7, MYST2), also interacts directly with p53 [17]. Unlike p300, HBO1 has not been reported to acetylate p53, even though it is involved in activating transcription of p53-responsive genes, including p21/CDKN1A [18]. HBO1 has also been shown to contribute to transcriptional activation through interactions with hormone nuclear receptors and AP-1 transcription factors [19–21]. Outside of its transcriptional functions, HBO1 helps modulate replication by serving as a coactivator for the replication licensing factor, CDT1 [22, 23]. In this context, HBO1 loading onto the chromatin promotes chromatin structure remodeling and subsequent recruitment of putative DNA helicase MCM2-7 [23].

Given the fundamental role of p53 in maintaining genome stability, in more than half of all cancers, it is functionally disabled through mutation [24]. In those cancer cells that retain wild-type p53, defects frequently occur in other components required for proper p53 function [6]. For example, multiple types of leukemia/lymphoma show a high frequency of mutations within the genes encoding p300 and CBP that abolish the HAT activities of these homologous proteins and prevent full acetylation of p53 [25–27]. Furthermore, tumor viruses have evolved mechanisms to inhibit p53 activity. One example is the complex retrovirus, human T-cell Leukemia Virus type 1 (HTLV-1), which is the etiologic agent of adult T-cell leukemia (ATL), a fatal malignancy characterized by uncontrolled proliferation of CD4^+^ T-cells [28]. While most ATL cells express wild-type p53 [29, 30], the function of the tumor suppressor is consistently impaired [31]. This effect has been attributed to the HTLV-1-encoded protein, Tax [32], which has been reported to inhibit p53 activity either by stimulating NF-κB signaling or by sequestering p300/CBP from p53, or through a separate, undefined mechanism [33–36]. In lieu of these reports, ATL cells from most patients do not express Tax due to deletion or methylation of the 5′ long terminal repeat (LTR) of the HTLV-1 provirus [37–39] which regulates expression of the *tax* gene and all other viral genes with the exception of *hbz* [28]. The *hbz* gene is consistently expressed in ATL cells [40, 41], as it is encoded on the negative strand of the provirus and regulated by a promoter in the 3′ LTR that does not undergo the same modifications as the 5′ LTR [28, 42]. This gene encodes the nuclear protein, HTLV-1 basic leucine zipper (bZIP) factor (HBZ) [42].

We previously found that HBZ interacts with multiple domains of p300/CBP, including the HAT domain [43]. The binding of HBZ to the HAT domain inhibits its enzymatic activity, which reduces p53 acetylation following induction of DNA damage [44]. In the current study, we evaluate the effect of HBZ on p53 transcriptional activity. Using HCT116 cells, in which the p53 signaling pathway is intact, we found that HBZ reduces transcription of the p53-responsive genes, p21/CDKN1A and GADD45A, which contribute to cell cycle arrest. Mechanistically, this effect occurs through inhibition of the HAT activities of both p300 and HBO1. Functionally, this effect delays the onset of G2/M arrest induced by etoposide. These results indicate that HBZ contributes to the loss of function of p53 observed during HTLV-1 infection and maintains p53 in an inactive state in ATL cells lacking other viral proteins.

## RESULTS

### HBZ inhibits p53 transcriptional activity on specific genes

**Figure 1:**
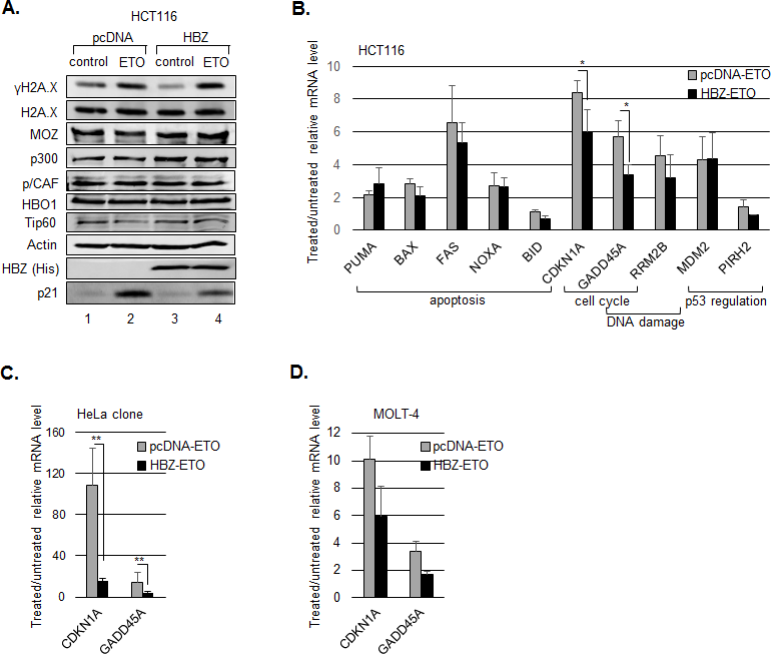
HBZ inhibits p53-mediated activation of p21/CDKN1A and GADD45A HCT116 p53^+/+^ cells were transiently transfected with an HBZ or empty expression vector and treated with etoposide (ETO) or the DMSO vehicle control (control) for 8 hours. **A.** Expression of HAT proteins. Nuclear extracts were analyzed by Western blot using the antibodies indicated. **B.** mRNA levels of p53-responsive genes. The graph shows real-time PCR data averaged from three or more independent experiments ± S.D. **C.** mRNA levels of p21/CDKN1A and GADD45A genes. HeLa cells stably transfected with an HBZ or empty expression vector and treated with etoposide (ETO) or the DMSO vehicle control (control) for 6 hours. The graph shows real-time PCR data averaged from four independent experiments ± S.D. *P < 0.05; **P < 0.005 (two-tailed Student *t* test). **D.** p21/CDKN1A and GADD45A mRNA levels from MOLT-4 cells transduced with pBABE-GFP-HBZ or pBABE-GFP. The graph shows real-time PCR data averaged from two independent experiments ± S.D.

We previously showed that HBZ inhibits p53 acetylation by the homologous coactivators, p300 and CBP [44]. Given that this modification contributes to the transcriptional activity of p53 following DNA damage [16], it was possible that HBZ repressed expression of genes activated by p53. To test this hypothesis, we analyzed expression of p53-responsive genes in HCT116 cells that express wild type p53 (p53^+/+^) and are commonly used to study the p53 pathway. In addition to p300 and CBP, other HAT-containing proteins acetylate p53 [16], and using western blot analysis, we confirmed that these proteins are expressed in the HCT116 cell line (Figure 1A). To examine potential effects of HBZ on expression of p53-responsive genes, we transfected cells with either an empty vector or an expression vector for the HBZ splice 1 isoform (herein referred to as HBZ), which is the predominant isoform expressed in ATL cells [41, 45]. Etoposide was then used to induce p53 acetylation and activation [8], and RNA was extracted and subjected to quantitative RT-PCR analysis. The p53-responsive genes tested included those involved in cell cycle inhibition (p21/CDKN1A, GADD45A), regulation of p53 (MDM2, PIRH2), the DNA damage response (GADD45, RRM2B), and induction of apoptosis (BID, FAS, PUMA, NOXA, BAX) [46]. As expected, etoposide treatment increased H2A.X phosphorylation, consistent with the induction of DNA damage (Figure 1A), and activated transcription of most of the p53-reponsive genes (Figure 1B). While a general trend was observed in which cells expressing HBZ exhibited a reduction in the mRNA levels of the genes tested, this effect was only significant for p21/CDKN1A and GADD45A. Consistent with this effect, HBZ also decreased p21/CDKN1A protein abundance following etoposide treatment (Figure 1A). In untreated cells, HBZ did not reduce the basal expression of these genes (data not shown), which is the same result found with mouse T-cells transiently expressing HBZ [47]. These results suggest that HBZ affects the expression of only a subset of p53-responsive genes with overlapping functions in cell cycle inhibition. We also confirmed that etoposide treatment did not affect the nuclear levels of the HATs (Figure 1A, lanes 2 and 4). In these experiments the transfection efficiency was estimated to be approximately 50%. Consequently, the reduction in p21/CDKN1A and GADD45A mRNA was more pronounced when comparing a HeLa clonal cell line stably expressing HBZ to a clonal cell line carrying the empty vector (Figure 1C).

Considering that the oncogenic effects caused by HTLV-1 infection arise in a T-cell environment, we tested whether HBZ also reduced the extent of p21/CDKN1A and GADD45A activation in the MOLT-4 T-cell line. While the status of p53 appears to vary among different sources of this line [48, 49], the cells we tested express the p53 protein [50, 51] and display activation of p21/CDKN1A and GADD45A expression following etoposide treatment. In analyzing cells transduced with HBZ versus cells transduced with the empty expression vector, we observed a smaller increase in the levels of p21/CDKN1A and GADD45A mRNA in the presence of HBZ (Figure 1D), consistent with results obtained using HCT116 and HeLa cells. As with the transfected HCT116 cells, the modest effect of HBZ may be due to a limited transduction efficiency.

### HBZ inhibits p21/CDKN1A transcription by inhibiting p300 and HBO1 activity

**Figure 2:**
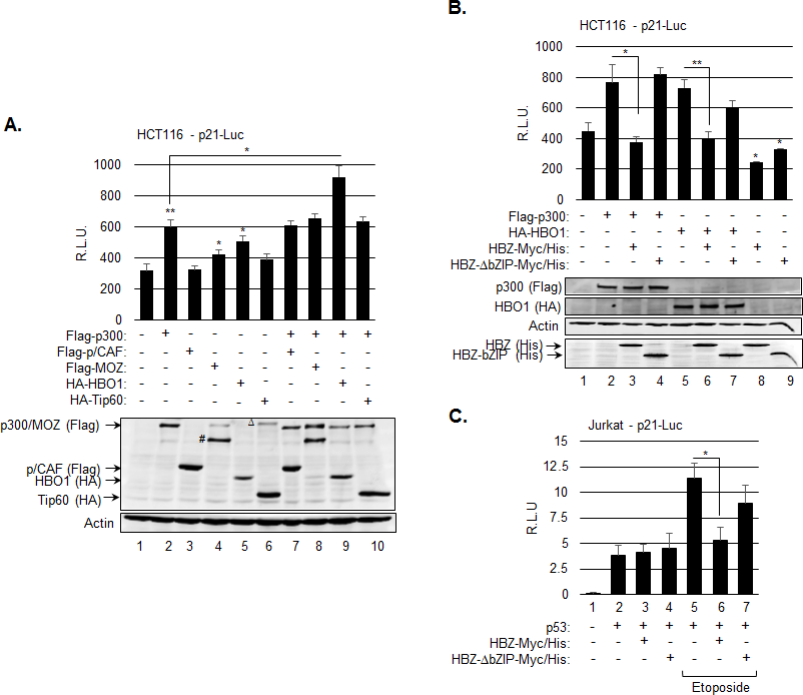
HBZ represses transcription from the p21/CDKN1A promoter through inhibition of p300 and HBO1 **A.** p300 and HBO1 produce an additive increase in transcription from the p21/CDKN1A promoter. HCT116 p53^+/+^ cells were cotransfected with p21-luc (200 ng), and expression vectors for Flag-p300 (400 ng), Flag-p/CAF (50 ng), Flag-MOZ (400 ng), HA-HBO1 (400 ng), or HA-Tip60 (50 ng) as indicated. The graph shows average luminescence values ± S.D. from one experiment performed in triplicate and is representative of three independent experiments. Cellular lysates used for the luciferase assay were also analyzed by Western blot using the antibodies indicated. # denotes a crossreactive protein or truncated product; Δ denotes a crossreactive protein. **B.** HBZ inhibits p300- and HBO1-mediated activation of transcription from the p21/CDKN1A promoter. HCT116 p53^+/+^ cells were cotransfected with p21-luc (200 ng) and expression vectors for Flag-p300 (400 ng), HA-HBO1 (400 ng), HBZ-Myc-His and/or HBZ-ΔbZIP-Myc-His as indicated. The graph shows average luminescence values ± S.D. from one experiment performed in triplicate and is representative of three independent experiments. Cellular lysates used for the luciferase assay were also analyzed by Western blot using the antibodies indicated. *P < 0.05; **P < 0.005 (two-tailed Student *t* test). **C.** HBZ inhibits etoposide-mediated activation of transcription from p21/CDKN1A promoter in T-cells. Jurkat T-cells were cotransfected with p21-luc (200 ng) and expression vectors for p53 (25 ng), HBZ-Myc-His and/or HBZ-Δb ZIP-Myc-His (800 ng) as indicated. Cells were treated with etoposide for 5 hours prior to harvesting. The graph shows average luminescence values ± S.D. from one experiment performed in triplicate and is representative of two independent experiments. *P < 0.05 (two-tailed Student *t* test).

We next examined whether an inhibitory effect of HBZ on one or more of the HAT proteins reduces transcription from the p21/CDKN1A promoter. The HAT proteins p300, p/CAF (KAT2B), MOZ (KAT6A, MYST3) and Tip60 (KAT5) participate in the activation of p21/CDKN1A expression, in part through acetylation of p53 [16]. In addition, the HAT protein, HBO1, contributes to the activation of p21/CDKN1A expression through a p53-dependent mechanism, but has not been reported to acetylate p53 [18]. In experiments, we cotransfected HCT116 cells with an expression vector for each HAT protein and the WWP-Luc reporter construct, which contains the p21/CDKN1A promoter, encompassing the p53 responsive elements, upstream of the luciferase gene [2]. Ectopic expression of p300, MOZ and HBO1 led to a significant increase in luciferase activity, while expression of p/CAF and Tip60 did not (Figure 2A). We additionally analyzed luciferase activity in cells ectopically expressing both p300 and one of each of the other HAT proteins. This approach was used to test whether p300 modulates the effects of other HAT proteins at the p21/CDKN1A promoter, as p300 has been shown to regulate acetylation of the FoxP3 transcription factor by Tip60 [52]. Interestingly, only expression of HBO1 with p300 increased the level of activation beyond that of p300 alone (Figure 2A). This increase appeared to be additive, suggesting that these two HATs have distinct functions in the regulation of p21/CDKN1A expression.

Based on these results, we performed additional reporter assays to test whether HBZ inhibits the effects of p300 and HBO1 at the p21/CDKN1A promoter. Interestingly, co-transfection of the HBZ expression vector prevented both HAT proteins from activating luciferase transcription (Figure 2B, lanes 3 and 6). We also analyzed the effects of a bZIP deletion mutant of HBZ (HBZ–ΔbZIP) based on previous data showing that the bZIP domain of HBZ inhibits p300 HAT activity through a direct interaction with the HAT domain [44]. In contrast to full-length HBZ, HBZ-ΔbZIP did not block activation from the promoter by either p300 or HBO1 (Figure 2B, lanes 4 and 7). In the absence of exogenous p300 and HBO1, both HBZ and HBZ-ΔbZIP caused a reduction in luciferase activity. This effect was not investigated further, but may arise from the interaction between HBZ and the KIX domain of p300 [43].

**Figure 3:**
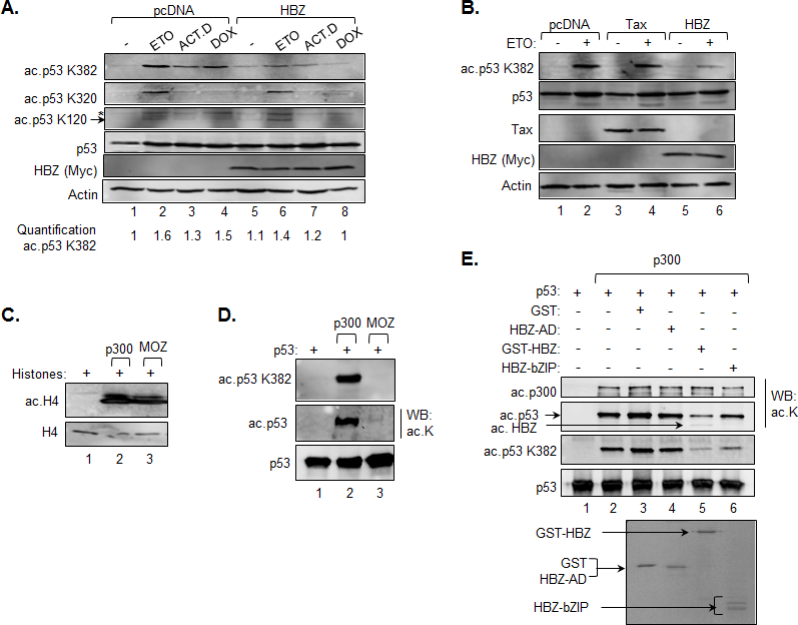
HBZ inhibits acetylation of p53 K382 **A.** and **B.** HCT116 p53^+/+^ cells were transiently transfected with an HBZ, Tax or an empty expression vector and, 48 h post-transfection, were treated with etoposide (ETO), actinomycin D (ACT.D), doxorubicin (DOX) or the DMSO vehicle control (−) for 8 hours as indicated. Nuclear extracts were prepared and analyzed by Western blot using the antibodies indicated. **C.** Acetylation of histone H4 by p300 and MOZ. *In vitro* HAT assays were performed using recombinant histones (2 μM), p300 (2 nM) and MOZ-HAT (0.15 μM) and analyzed by Western blot using antibodies against histone H4 and acetylated histone H4 as indicated. **D.** Acetylation of p53 by p300 and MOZ. *In vitro* HAT assays were performed using the same concentrations of recombinant proteins as above, but with p53 (0.1 μM) replacing histones as the substrate. Reactions were analyzed by western blot using antibodies against acetylated lysine, p53 acetyl-K382 and p53. **E.** HBZ inhibits acetylation of p53 by p300. *In vitro* HAT assays were performed using recombinant p300 (2 nM), p53 (25 nM) and supplemented with GST (0.3 μM), GST-HBZ (0.3 μM), HBZ-AD (0.3 μM) or HBZ-bZIP (0.3 μM) where indicated. Reactions were analyzed by Western blot using antibodies against acetylated lysine, p53 acetyl-K382 and p53. Identical quantities from the same batch of proteins used in the HAT assay were resolved by SDS-PAGE and stained with Coomassie (lower panel).

We also examined the effects of HBZ on transcription from the p21/CDKN1A promoter in Jurkat T-cells. In reporter assays, cells were co-transfected with a p53 expression plasmid, as Jurkat cells lack endogenous p53 expression [53, 54]. Interestingly, HBZ did not affect the level of basal transcription induced by p53 in the absence of stimulation (Figure 3C, lanes 3 and 4). However, we found that etoposide-mediated activation of transcription from the p21/CDKN1A promoter was abrogated in presence of HBZ, but was retained in the presence of HBZ-ΔbZIP (Figure 3C, lanes 6 and 7). These observations are consistent with the results obtained using HCT116 cells and indicate that HBZ is able to inhibit p53 transcriptional activity in a T-cell environment.

### HBZ inhibits acetylation of p53 at lysine K382 by p300/CBP

We previously showed that HBZ reduces p53 K382 acetylation in HeLa cells [44]. These cells are transformed by human papilloma virus 18 and therefore express the viral protein E6 which also inhibits p53 activity [55]. To confirm that HBZ is able to reduce p53 K382 acetylation independent of this other viral protein, we transfected the HCT116 cells with the HBZ expression vector and subsequently treated the cells with etoposide or other genotoxic agents to induce p53 acetylation [12, 56]. Consistent with our previous results, we observed a reduction in p53 K382 acetylation in the presence of HBZ (Figure 3A). However, HBZ did not affect acetylation of K320, which is a substrate for p/CAF [57], and K120, which is a substrate for Tip60, MOF and MOZ [58–60] (Figure 3A).

To verify that the reduction in K382 acetylation was a specific effect of HBZ, we tested whether another HTLV-1-encoded protein, Tax, affected the level of acetylation of this residue. Tax has been reported to repress p53 activity through multiple mechanisms that do not directly involve p53 acetylation [33–36]. As expected, expression of Tax in HCT116 cells treated with etoposide did not lead to an alteration in K382 acetylation (Figure 3B), suggesting that HBZ regulates p53 activity in a distinct manner from that of Tax.

Given that both p300/CBP and MOZ have been reported to acetylate K382 of p53 [9, 60], we were interested in determining which HAT protein is targeted by HBZ. In order to differentiate between p53 acetylation by p300 and MOZ, we performed *in vitro* HAT assays using recombinant proteins. We found that both p300 and MOZ acetylated histones, indicating that both proteins were enzymatically active (Figure 3C). However, only p300 acetylated p53 at K382 (Figure 3D, top panel). As this result was unexpected, we used a pan-acetyl-lysine antibody in the assay and detected weak p53 acetylation by MOZ in comparison to strong acetylation by p300, which is known to acetylate at least eight lysine residues [16] (Figure 3D, middle panel). This observation confirms acetylation of p53 by MOZ, suggesting that in our *in vitro* assays, acetylation of p53 by MOZ is restricted to K120. Consistent with our previous study [44], addition of full-length HBZ or the bZIP domain of HBZ reduced K382 acetylation by p300, while addition of the activation domain of HBZ did not affect acetylation (Figure 3E). As previously observed, HBZ was acetylated in the reaction [44]. These data suggest that inhibition of p300 HAT activity by HBZ is responsible for the reduction in p53 K382 acetylation in the HCT116 cells, which contributes to HBZ-mediated repression of transcription from the p21/CDKN1A promoter.

### HBO1 is a coactivator of p53 transcription

**Figure 4:**
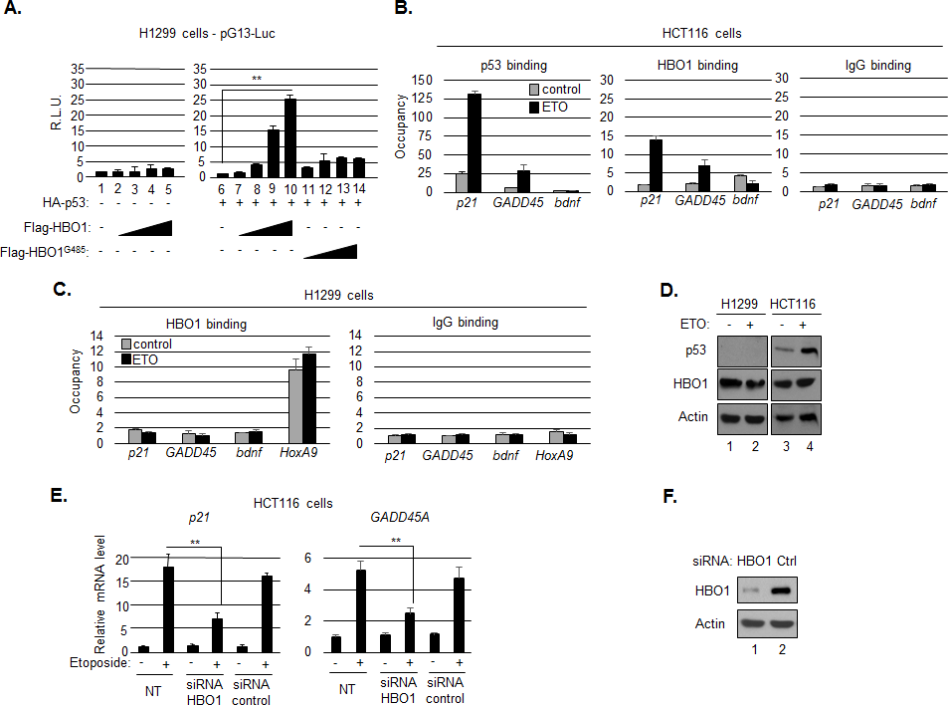
HBO1 contributes to p53-mediated activation of p21/CDKN1A **A.** HBO1 augments p53-dependent transcription. H1299 cells were cotransfected with pG13-luc (150 ng), and expression vectors for HA-p53 (200 ng), Flag-HBO1 (increasing concentration up to 1 μg), Flag-HBO1^G485^ (increasing concentration up to 1 μg), or a combination of those as indicated. The graph shows relative luminescence values ± S.D. from a triplicate experiment. Data are normalized to the control condition artificially set to 1 (lane 1 for the graph on the left and lane 6 for the graph on the right). **B.** Etoposide induces HBO1 recruitment to the p21/CDKN1A and GADD45A promoters. Chromatin immunoprecipitation (ChIP) analysis was performed on chromatin prepared from untreated HCT116 p53^+/+^ cells (grey bars) or cells treated with etoposide (black bars) using p53 and HBO1 antibodies or a preimmune serum (IgG). Precipitated DNA fragments were subjected to real-time PCR analysis with primers amplifying the BNDF promoter, the p53 binding region in the p21/CDKN1A promoter and the GADD45A promoter. Data are presented as fold enrichment over a control unrelated regions. **C.** HBO1 recruitment to the p21/CDKN1A and GADD45A promoters is dependent on p53. ChIP analysis in H1299 cells untreated (grey bars) or treated with etoposide (black bars) using an HBO1 antibody or a preimmune serum (IgG). Precipitated DNA fragments were subjected to real-time PCR analysis with primers amplifying the BNDF promoter, the p53 binding region in the p21/CDKN1A promoter, the GADD45A promoter and an HBO1 binding site in the HOXA9 promoter. **D.** Characterization of H1299 and HCT116 p53^+/+^ cells used in the study by Western blot. **E.** HBO1 depletion reduces transcription of p21/CDKN1A and GADD45A induced by etoposide. RT-PCR analyses of p21/CDKN1A and GADD45A expression were performed on mRNAs prepared from HCT116 cells transiently transfected with a specific siRNA against HBO1 or a control siRNA and further treated with ETO or not. Expression in each condition is normalized to the condition: -ETO, NT. **F.** The efficiency of the siRNA was estimated by Western blot.

In addition to affecting p300, HBZ also blocked the effects of HBO1 at the p21/CDKN1A promoter. In order to define the mechanism of inhibition, it was important to first determine the function of HBO1 at the p21/CDKN1A promoter. Previously, HBO1 was found to be associated with the p21/CDKN1A promoter, and shRNA-mediated knockdown of p53 reduced transcriptional activation by HBO1, suggesting that HBO1 activity was dependent on p53 [18]. However, in a separate study, p53 was found to bind to, and inhibit the HAT activity of HBO1 [17]. Consequently, we first tested whether HBO1 positively or negatively affected p53 transcriptional activity from a synthetic p53 responsive reporter plasmid (pG13-Luc), using the p53-negative cell line, H1299. As expected, transfection of increasing amounts of an HBO1 expression vector did not activate transcription in the absence of p53 (Figure 4A). However, co-transfection of a p53 expression vector led to a dose-depend increase in transcription in response to HBO1, an effect that was abrogated by substituting wild-type HBO1 with a HAT-defective mutant, HBO1^G485^ [17, 61] (Figure 4A).

Based on these results, we used chromatin immunoprecipitation (ChIP) assays to test whether p53 facilitates recruitment of HBO1 to the p21/CDKN1A and GADD45A promoters. In HCT116 cells, p53 activation by treatment with etoposide increased the level of enrichment of both p53 and HBO1 at both promoters (Figure 4B). This effect was not due to an increase in HBO1 expression (Figure 4D). In contrast to the p21/CDKN1A and GADD45A promoters, the signal for both proteins remained close to background at the BDNF promoter, which is not regulated by p53 (Figure 4B). In H1299 cells, HBO1 was enriched at the HoxA9 promoter, as we showed previously [23], but not at the p21/CDKN1A and GADD45A promoters, regardless of etoposide treatment (Figure 4C).

We also tested whether HBO1 activated endogenous expression of p21/CDKN1A and GADD45A. Using HCT116 cells we found that treatment with etoposide caused an increase in the level of p21/CDKN1A and GADD45A mRNA; however, this increase was significantly reduced by siRNA-mediated knockdown of HBO1 expression (Figure 4E). Overall, the results above indicate that HBO1 is recruited to the p21/CDKN1A and GADD45A promoters where it participates in the activation of p53-mediated transcription *via* its HAT activity.

### HBZ inhibits HBO1 HAT activity

**Figure 5:**
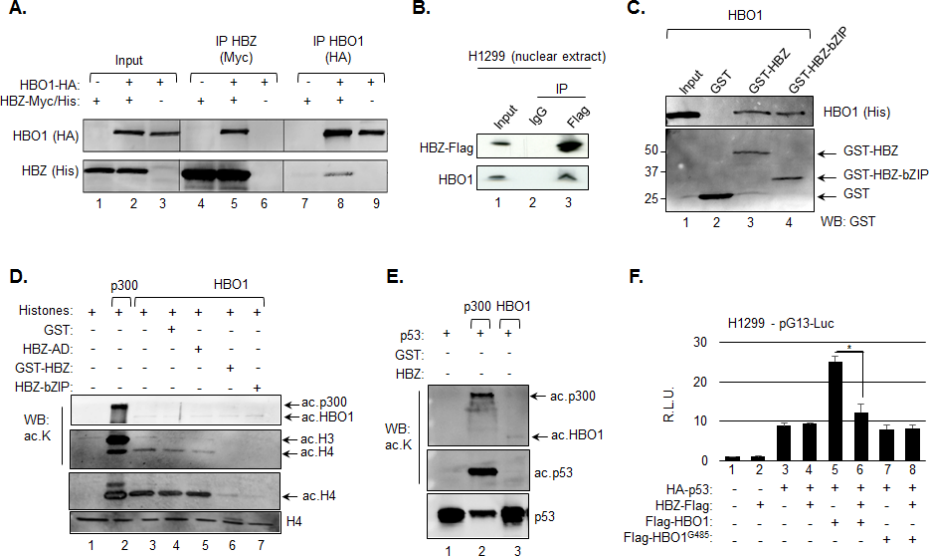
HBZ inhibits HBO1 HAT activity **A.** HBZ and HBO1 interact *in vivo*. Whole cell extracts were prepared from HEK293T/17 cells transfected with expression vectors for HBZ-Myc-His and/or HBO1-HA (6 μg of each vector). Extracts were analyzed by immunoprecipitation (IP) and Western blotting using Myc and HA epitope antibodies as indicated. Whole cell extracts (10% of the IP inputs) are shown in lanes 1 to 3. **B.** HBZ interacts with endogenous HBO1. Nuclear extract was prepared from H1299 cells transfected with an expression vector for HBZ-Flag (3 μg) and analyzed by IP using preimmune serum (IgG) or a Flag epitope antibody as indicated. The Western blot was probed with Flag and HBO1 antibodies. The nuclear extract (20% of the IP input) is shown in lane 1. **C.** HBZ and HBO1 interact directly. Recombinant HBO1 (10 pmol) was incubated with 25 pmol of GST, GST-HBZ or GST-bZIP. Bound proteins were detected by Western blot using 6xHis and GST antibodies. A fraction of the HBO1 input (10%) is shown in lane 1. **D.** HBZ inhibits HBO1 HAT activity. *In vitro* HAT assays were performed using recombinant histones (2 μM), p300 (2 nM), HBO1 (18 nM), GST (0.2 μM), GST-HBZ (0.2 μM), HBZ-AD (0.2 μM) and HBZ-bZIP (0.2 μM) as indicated and analyzed by Western blot using antibodies against acetylated lysine, acetylated histone H4 and histone H4 as indicated. **E.** HBO1 does not acetylate p53. *In vitro* HAT assays were performed using the same concentrations of the indicated recombinant proteins as above, but with p53 (0.1 μM) replacing histones as the substrate. Reactions were analyzed by Western blot using antibodies against acetylated lysine and p53. Only a portion of the HAT assay was loaded in lane 2. **F.** HBZ inhibits HBO1-mediated activation of the p21/CDKN1A promoter. H1299 cells were cotransfected with pG13-luc (150 ng), and expression vectors for HA-p53 (200 ng), Flag-HBZ (150 ng), Flag-HBO1 (200 ng), Flag-HBO1^G485^ (200 ng), or combination of those as indicated. The graph shows relative luminescence values ± S.D. from a triplicate experiment. Data are normalized to the control condition (lane 1) artificially set to 1. *P < 0.05 (two-tailed Student *t* test).

Previous results from a yeast two-hybrid analysis revealed that HBO1 interacts with HBZ [62]. To confirm this finding in mammalian cells, we co-transfected HEK293T/17 cells with expression vectors for HBO1 and HBZ and found that each protein was coimmunoprecipitated with the other (Figure 5A). In addition, in H1299 cells, endogenous HBO1 was coimmunoprecipitated with ectopically expressed HBZ (Figure 5B). Together, these results suggest that HBZ and HBO1 interact in a p53-independent manner. Using a GST pull-down assay, we showed that GST fused to full-length HBZ or the bZIP domain alone bound directly to HBO1 (Figure 5C). These results indicate that the direct interaction between HBZ and HBO1 is, in part, mediated through the bZIP domain of the viral protein.

Considering that the bZIP domain of HBZ is sufficient to inhibit p300 HAT activity through a direct interaction with the coactivator [44], it was possible that HBZ also inhibited the HAT activity of HBO1. To test this hypothesis, we performed *in vitro* HAT assays using recombinant histones as substrates. In comparison to p300, HBO1 HAT activity was weaker and favored histone H4 (Figure 5D, lanes 2 and 3). Consistent with our hypothesis, both full-length HBZ and the bZIP domain alone inhibited HBO1 HAT activity, while the activation domain of HBZ did not (Figure 5D, lanes 5–7). Unlike histones H3 and H4, p53 was not acetylated by HBO1 (Figure 5E).

Using H1299 cells and the pG13-Luc reporter plasmid we then tested whether HBZ inhibits p53 transcriptional activity augmented through HBO1. In the absence of HBZ, p53 increased luciferase activity, which was further elevated by ectopic expression of HBO1, but not by HBO1^G485^ (Figure 5F, lanes 1, 3, 5 and 7). Consistent with a previously study [63], HBZ did not affect the activation of transcription by p53 alone (Figure 5F, lane 4); however, HBZ did inhibit the activation of transcription coordinated through p53 and HBO1 together (Figure 5F, lane 6).

**Figure 6:**
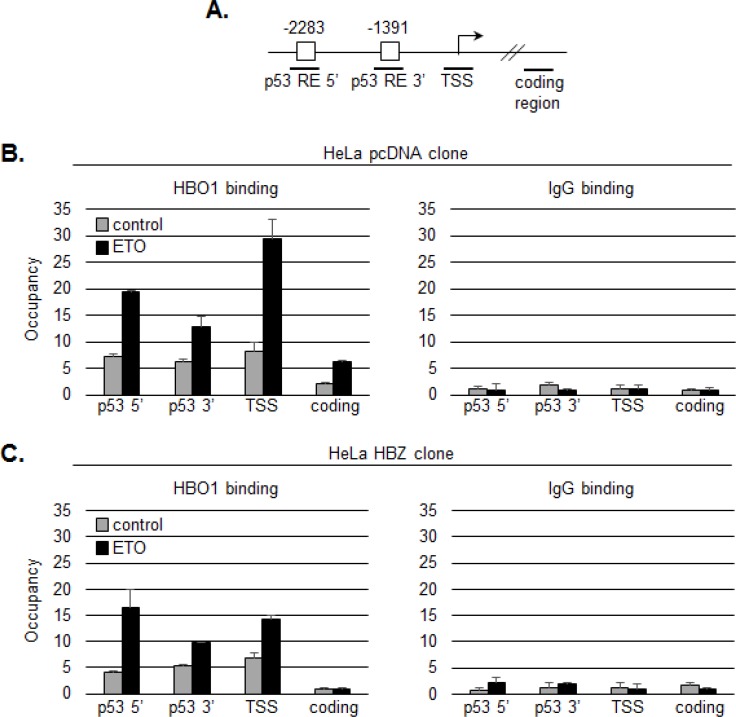
HBZ inhibits binding of HBO1 to the p21/CDKN1A promoter following etoposide treatment **A.** Graphic representation of the p21/CDKN1A promoter showing 5′ and 3′ p53 responsive elements (RE, white boxes) and the transcription start site (TSS). Bold horizontal lines denote real-time PCR amplicons. ChIP analyses were performed on chromatin prepared from untreated (grey bars) or etoposide-treated (black bars) cells using an HBO1 antibody or a preimmune serum (IgG). Precipitated DNA fragments were subjected to real-time PCR analysis. Data are presented as fold enrichment over a control unrelated regions. **B.** Analysis of a HeLa clonal cell line containing the empty pcDNA 3.1 vector. **C.** Analysis of a HeLa clonal cell line stably expressing HBZ.

While the effect on transcription is expected to stem from the inhibition of HBO1 HAT activity, it was possible that HBZ additionally influenced the recruitment of HBO1 to the p21/CDKN1A promoter. To test this hypothesis, we used ChIP assays to compare levels of HBO1 enrichment at the promoter in a HeLa clonal cell line expressing HBZ and one carrying the empty vector [64]. We analyzed amplicons corresponding to the p53 responsive elements in the promoter, the transcription start site and a distal coding region (Figure 6A). In absence of HBZ, etoposide treatment led to enrichment of HBO1 at the distal and proximal p53 responsive elements, and more substantially, at the transcription start site as previously reported (Figure 6B, [18]). In comparison, cells expressing HBZ exhibited a lower level of HBO1 enrichment at the transcription start site following etoposide treatment (Figure 6C). These results suggest that HBZ, in addition to inhibiting the HAT activity, may also interfere with the recruitment of HBO1 to the promoter in proximity of the transcription start site.

### HBZ delays cell cycle arrest induced by etoposide treatment

**Figure 7:**
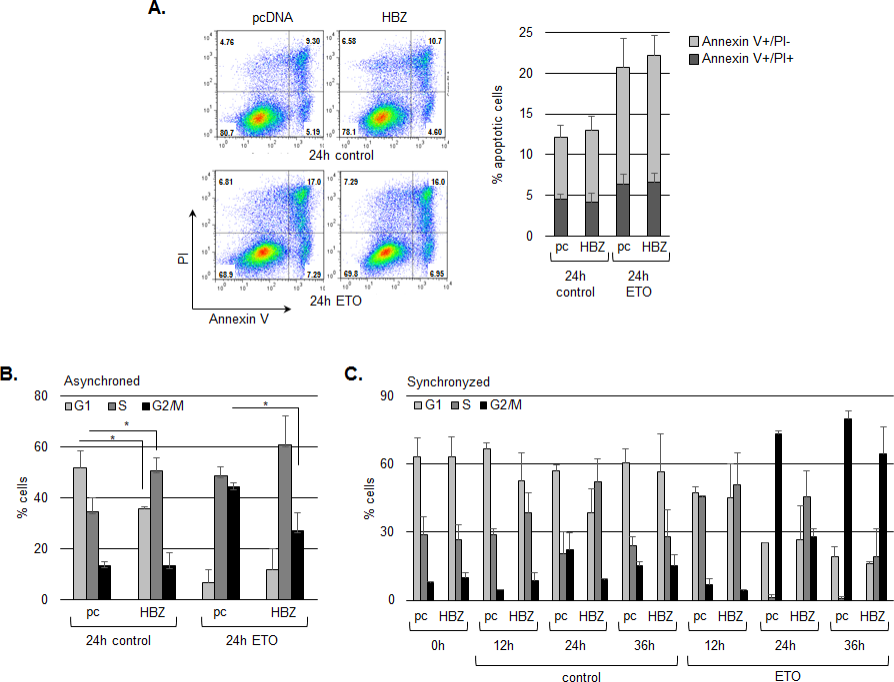
HBZ delays cell cycle arrest in G2/M following treatment with etoposide **A.** HBZ does not alter the level of apoptosis induced by etoposide. HCT116 p53^+/+^ cells were transiently transfected with an HBZ or empty expression vector and, 48 h post-transfection, were treated with etoposide (ETO) or the DMSO vehicle (control) for 24 hours as indicated. Plots are from a representative experiment and the bar graph shows the average flow cytometry data of early apoptosis (grey bars) and late apoptosis (darker grey bars) from three independent experiments ± S.D. **B.** HBZ decreases G2/M arrest in an asynchronous cell population. HCT116 p53^+/+^ cells were transiently transfected with an HBZ or the empty expression vector and, 48 h post-transfection, were treated with etoposide (ETO), or the DMSO vehicle (control) for 24 hours as indicated. The graph shows the average flow cytometry data from three independent experiments ± S.D. *P < 0.05 (two-tailed Student *t* test). **C.** HBZ delays G2/M arrest in a synchronized cell population. Transfected HCT116 p53^+/+^ cells were arrested in G1 phase by serum starvation and treated with etoposide (ETO), or the DMSO vehicle (control) for the times indicated. The graph shows the average flow cytometry data from two independent experiments ± S.D.

Our data indicate that HBZ does not affect p53-mediated activation of pro-apoptotic genes and does not alter levels of p53 K120 acetylation, which is associated with induction of apoptosis. To extend these findings, we compared apoptotic cell populations between HCT116 cells transfected with the HBZ or the empty expression vector following etoposide treatment. As expected, Annexin V and/or propidium iodide staining did change significantly in the presence of HBZ, supporting that HBZ does not suppress induction of apoptosis by p53 (Figure 7A). It is important to note that, in these experiments, dead cells were removed from cultures just prior to the addition of etoposide. Indeed, while the HBZ protein has also been reported to attenuate apoptosis [65], results from one study have shown that the HBZ protein induces apoptosis [66].

Finally, we were interested in determining whether inhibition of p21/CDKN1A expression by HBZ is associated with a reduction in p21/CDKN1A function. p21/CDKN1A induces cell cycle arrest at the G1/S [2, 3, 67] and G2/M checkpoints [68–73]. In addition, GADD45A also promotes G2 arrest [74–76], and as shown in Figure 1, its expression is reduced by HBZ following etoposide treatment. To test whether HBZ influences the G2/M checkpoint, we analyzed the effects of the viral protein on etoposide-mediated cell cycle arrest in HCT116 cells. In asynchronous cells, HBZ appeared to reduce the sensitivity of cells to etoposide following 24 hours of treatment, as less HBZ-expressing cells occupied G2 phase compared to cells transfected with the empty expression vector (Figure 7B). However, it was possible that this effect was due to a difference in proliferation between HBZ and empty vector cells, as HBZ has been shown to increase T-cell proliferation [40, 77]. Therefore, to show that results were specifically due to a difference in cell cycle arrest, we repeated experiments using synchronized cells and evaluated the effects of etoposide at different time points. Following 24 hours of etoposide treatment, we again observed less HBZ-expressing cells in G2 compared with cells carrying the empty vector; however at 36 hours of treatment, the HBZ-expressing cells appeared to arrest in G2 (Figure 7C). Together, the results above indicate that HBZ delays G2/M arrest induced by DNA damage.

## DISCUSSION

In this study we provide evidence that HBZ reduces p53-mediated activation of the cell cycle arrest genes, p21/CDKN1A and GADD45A, following etoposide treatment. Analysis of the p21/CDKN1A promoter in reporter assays corroborated that repression by HBZ occurred at the level of transcription and involved repression of the HAT activities of p300 and HBO1.

In the case of p300, HBZ inhibited its ability to directly acetylate p53. In addition to acting as a coactivator for p53, p300 acetylates multiple lysine residues in p53, including those in the C-terminal domain (K370, K372, K373, K381 and K382) that, when acetylated, enhance the DNA binding activity of p53 [9]. We found that expression of HBZ consistently dampened K382 acetylation of p53 in response to various DNA damaging agents. In contrast, HBZ did not appear to inhibit the HAT activities of other proteins known to modify p53. Specifically, HBZ did not appear to affect acetylation of K120, which is targeted by either Tip60, MOF or MOZ [58–60], nor did it appear to affect acetylation of K320, which is a substrate for p/CAF [57]. In addition to p300, MOZ has been reported to acetylate p53 at K382 [60]. We confirmed that HBZ represses K382 acetylation by p300 in *in vitro* HAT assays [44]. However, we were unable to detect K382 acetylation by MOZ in these assays despite the ability of the recombinant, purified polypeptide to acetylate histone proteins. Considering that we tested a truncated form of MOZ (amino acids 497–780), encompassing the HAT and acidic domains, it is possible that the p53 interaction is mediated through a separate region of MOZ. Nevertheless, we speculate that K382 acetylation in HCT116 cells occurs primarily through p300 given that p300 elicited a significantly greater increase in transcription from the p21/CDKN1A promoter than MOZ in luciferase assays.

Interestingly, HBZ also repressed transcription from the p21/CDKN1A promoter through inhibition of HBO1. Recently, HBO1 was shown to localize to the p21/CDKN1A promoter and activate transcription in a p53-dependent manner [18]. This effect is likely to involve a direct interaction between HBO1 and p53 that was separately characterized [17]. Indeed, we observed that HBO1 is recruited to the p21/CDKN1A promoter in a p53-dependent manner and that the ability of HBO1 to augment transcriptional activity of p53 requires the functional HAT domain. Our results indicate that HBZ interferes with both processes given that HBZ inhibited HBO1 HAT activity *in vitro* and caused a reduction in the level of HBO1 associated with the promoter in cells. As with p300, the bZIP domain of HBZ interacts directly with HBO1 and is responsible for the inhibition of HAT activity. It is intriguing that HBZ is able to inhibit the HAT activities of both p300 and HBO1 considering the divergence in the amino acid sequences between their HAT domains. Future studies will determine whether HBZ targets an amino acid motif that is shared between these two proteins and explore the possibility that HBZ exhibits similar inhibitory effects on other HAT proteins.

**Figure 8:**
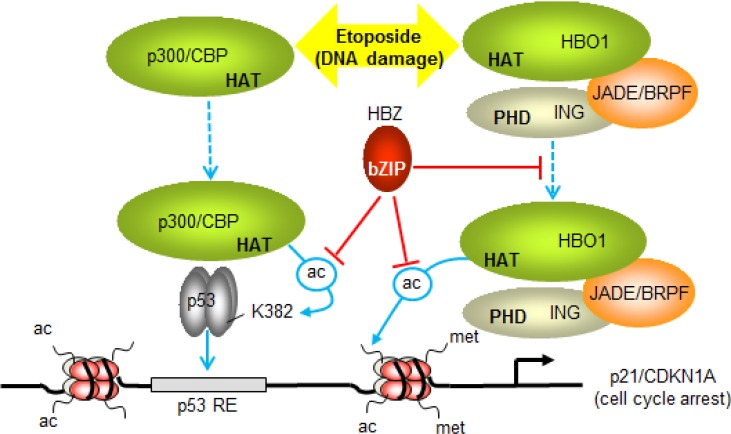
Model summarizing the effects of HBZ on p53-regulated transcription of p21/CDKN1A Etoposide-induced DNA damage, stimulates p53-dependent recruitment of p300 and HBO1 to the promoter (dashed blue arrows). During this process p300 acetylates p53 at K382 (ac-labeled arrow) and other lysine residues to increase the DNA-binding activity of p53 (solid blue arrow). HBO1 recruitment may be facilitated by associated ING and JADE/BRPF proteins, which interact with methylated histone H3 through their PHD finger domains. These interactions may position HBO1 in proximity to the transcription start site, where it acetylates histones. By inhibiting the HAT domains of p300 and HBO1, HBZ represses acetylation of p53 and promoter-associated histones. HBZ also restricts the recruitment of HBO1, which may lead to further reduction in acetylation of histones at the promoter. The sum of the effects of HBZ is to dampen the level of activation of p21/CDKN1A (and GADD45A) transcription, leading to a delay in cell cycle arrest induced by etoposide.

The ability of HBZ to inhibit the HAT activities of both p300 and HBO1 is likely to affect separate events associated with p53-mediated transcription from the p21/CDKN1A promoter (Figure 8). Our data indicate that, by binding p300, HBZ inhibits acetylation of p53, thereby reducing the transcriptional activity of the tumor suppressor. In addition, HBZ may also affect the coactivator function of p300 at the p21/CDKN1A promoter [13, 78, 79]. Unlike p300, HBO1 has not been reported to acetylate p53 [59], which we confirmed in this study. Therefore, HBZ is likely to impede the ability of HBO1 to acetylate histones at the p21/CDKN1A promoter. Indeed, HBO1 serves as the HAT component of a protein complex consisting of JADE and ING proteins, in which it is responsible for acetylation of specific lysine residues in histones H3 and H4 [80]. It is possible that HBO1 and p300 cooperate in other nuclear processes. In *Drosophila* the concerted HAT activities of CBP and the HBO1 orthologue, Chameau (Chm), hyperacetylate nucleosomes at gene amplification origins, which is required in the development of follicle cells [81]. In mammalian cells, HBO1 and p300 regulate CDT1 function. p300 acetylates CDT1 to promote its accumulation while HBO1 interacts with CDT1 to support its ability to recruit the MCM helicase [23, 82]. It would thus be of interest to characterize the potential cooperative roles of HBO1 and p300/CBP in replication licensing and determine whether HBZ affects this process.

The effects of HBZ on the transcriptional response of p53 is consistent with HBZ specifically counteracting cell cycle arrest following etoposide treatment. For example, acetylation of p53 at K382, which was reduced by HBZ, potentially favors the activation of genes involved in cell cycle arrest, while acetylation of p53 at K120, which was not affected by HBZ, has been linked to apoptosis [83]. Consistent with this pattern, HBZ did not affect apoptosis, but HBZ did dampened the G2/M phase arrest induced by etoposide, which is expected to involve the reduction in p21/CDKN1A as well as GADD45A expression. Indeed, p21/CDKN1A contributes to cell cycle arrest in both the G1 and G2 phases of the cell cycle through its ability to bind to and inhibit multiple cyclin/CDK complexes (reviewed in [73]). GADD45A participates in cell cycle arrest during the G2/M checkpoint, by also binding to, and blocking activation of cyclin/CDK complexes [74–76].

In contrast to HBZ, another HTLV-1-encoded protein, Tax, activates p21/CDKN1A expression, an effect that is independent of p53 [84]. Tax exhibits oncogenic properties and is believed to be essential for the development of ATL [28, 84]. Therefore, effects of Tax observed in HTLV-1-infected T-cell lines are often recapitulated in ATL cells from patients, which would suggest the effect of HBZ on p21/CDKN1A expression does not relate to ATL. However, unlike HTLV-1 cell lines that express high levels of p21/CDKN1A, expression of this gene is low to undetectable in ATL cell lines and ATL patient samples [85]. Therefore, although activation of p21/CDKN1A expression by Tax may occur during the course of HTLV-1 infection, it does not appear to apply to ATL maintenance.

In addition to inducing cell cycle arrest following stress, p21/CDKN1A and GADD45 promote senescence [86], which is a process that is also associated with Tax [87]. One of the many functions of Tax is to stimulate NF-κB signaling that, when constitutively activated, leads to increased expression of p21/CDKN1A and the onset of cellular senescence [88]. Interestingly, HBZ inhibits NF-κB signaling [44, 89], and this function is reported to alleviate Tax-induced senescence [88]. Based on results from our current study, it is possible that HBZ also counteracts senescence by reducing levels of p21/CDKN1A and GADD45A. In addition to these effects, HBZ has also been reported to increase expression of hTERT, the catalytic subunit of human telomerase reverse transcriptase [90], which may support constitutive telomerase activity, allowing cells to bypass senescence [91, 92]. Therefore, HBZ potentially utilizes multiple pathways to prevent senescence. Overall, by bypassing DNA damage-induced cell cycle arrest and senescence, cells expressing HBZ may accumulate mutations that eventually contribute to the development of ATL.

## MATERIALS AND METHODS

### Cell culture and treatments

HCT116 p53^+/+^ cells [68] were cultured in McCoy's 5A medium, HEK293T/17 (ATCC) and HeLa-S3 clones [64] were cultured in DMEM, H1299 and MOLT-4 cells were culture in RPMI-1640 medium, and Jurkat cells were culture in IMDM. All media were supplemented with 10% fetal bovine serum, 100 U/mL penicillin, 50 mg/mL streptomycin, and 2 mM L-glutamine. Cells were incubated at 37°C in a humidified atmosphere containing 5% CO_2_. Cells were transfected using Turbofect (Thermo Fisher Scientific) and described by the manufacturer. Genotoxic drugs were used at the following concentrations: 50 μM etoposide (Sigma), 10 nM actinomycin D (Sigma), or 0.2 μg/mL doxorubicin (Sigma). siRNA targeting HBO1 and control siRNA were purchased from Life Technologies (# 4390824 and #12935–112).

### Plasmids

Empty vectors used in this study included pcDNA3.1-Myc-His A (Thermo Fisher Scientific), pGEX-2T (GE Healthcare). *E. coli* and mammalian HBZ expression vectors included GST-HBZ, GST-HBZ bZIP, HBZ-AD_1-122_, pcDNA-HBZ-Myc-His, pcDNA-HBZ-bZIP-Myc-His and have been described [43, 93]. HBZ-Flag was prepared by NotI/HindIII excision of the HBZ cDNA from pcDNA-HBZ-Myc-His and insertion of the fragment into the same sites in pCMV-3Tag-8 (Stratagene). pBABE-GFP-HBZ was prepared by digesting pcDNA-HBZ-bZIP-Myc-His with ScaI/PvuII and inserting the fragment extending from the CMV promoter to the BGH polyadenylation signal into the BamH1 site of pBABE-GFP that was a gift from William Hahn (Addgene plasmid # 10668). DNA ends were polished prior to ligation. Other plasmids used for transduction, pCL-Ampho and pHCMVG, have been described [94, 95]. Reporter plasmids included pRL-TK and pRL-SV40 from Promega, WWP-Luc (p21 promoter) [2] and PG13-luc (wt p53 binding sites), which was a gift from Bert Vogelstein (Addgene plasmid # 16442) [2]. *E. coli* and mammalian HBO1 expression vectors included His_6_-HBO1, Flag-HBO1, Flag-HBO1^G485^ (HAT inactive) and HA-HBO1, which have been described [22, 23, 96]. Other expression vectors used in this study included pCI-Flag-p/CAF, which was a gift from Yoshihiro Nakatani (Addgene plasmid # 8941) [97], pcDNA3-HA-p53, pC53-SN3 [98]; pSG-Tax, pRSETA-p53, pCI-Flag-p300, Flag-MOZ and pcDNA-HA-Tip60, which have been described [33, 99–102]. The MOZ HAT domain was PCR-amplified from MYST3 and cloned into the BamHI/EcoRI sites of pGEX-2T (GE Healthcare). MYST3 was a gift from Cheryl Arrowsmith (Addgene plasmid # 25181).

### Preparation of cellular extracts and western blot assays

Nuclear extracts were prepared from 5 × 10^5^ cells/ 10 cm plate transfected for 48 hours and then treated with drugs or carrier for 8 hours (HCT116) or 5 hours (HeLa). Extracts were prepared as described [44], using solutions additionally supplemented with Halt Phosphatase Inhibitor Cocktail (Thermo Scientific). Proteins from nuclear extracts were resolved by SDS-PAGE and analyzed by Western blot as described [44]. The primary antibodies used for protein detection were as follows: MOZ (39868) and H4 (39270) antibodies purchased from Active Motif; p300 (sc-584), p/CAF (sc-13124), p21 (sc-397) and p53 (sc-126) antibodies purchased from Santa Cruz Biotechnology; pan-acetyl lysine (9441), p53 K382 (2525) and H2A.X (7631) antibodies purchased from Cell Signaling; p53 K320 (06-1283), p53 K120 (ABE286), Myc (clone 4A6, 05–724), acetyl H4 (06–866), actin (clone C4, MAB1501) and ser139-H2A.X (05–636) antibodies purchased from EMD Millipore; 6 × His (ab9108) antibody purchased from Abcam; Flag (M2, F3165) and HA (clone HA-7, H3663) antibodies purchased from Sigma-Aldrich; Tip60 (GTX112198) and HBO1 (N2C1, GTX102041) purchased from Genetex; and Tax hybridoma (168B17–46–92) obtained from the NIH AIDS Research and Reference Reagent Program.

### RNA extraction, cDNA synthesis and quantitative real-time PCR

Cells were transfected and treated as described above. RNA was extracted using TRIzol Reagent (Thermo Fisher Scientific). cDNA was synthesized with random hexamers using the RevertAid kit (Thermo Fisher Scientific). Quantitative real-time PCR was performed and analyzed as described [64]. Primers used were as follows: UBE2D2 F: TGCCTG AGATTGCTCGGATCTACA, UBE2D2 R: ACTTCTGAG TCCATTCCCGAGCTA, RRM2B F: GAGGAGCTCAG TTCCCTCAG, RRM2B R: TTCGTTGGTGTCTGAAG ATGA, BID F: GAGGAGCACAGTGCGGAT, BID R: GG AACCGTTGTTGACCTCAC, CDKN1A F: ACCATGTG GACCTGTCACTGCTT, CDKN1A R: AGAAGATGT AGAGCGGGCCTTGA, NOXA F: TGCAGGACTGT TCGTGTTCAGCTC, NOXA R: AGTAGCACACTCGAC TTCCAGCTCT, GADD45A F-a: GTGCTGGTGACGAAT CCACATTCA, GADD45A R-a: TGCCATCACCGTTCA GGGAGATTA, GADD45A F-b: GAGAGCAGAAGACC GAAAGGA, GADD45A R-b: CACAACACCACGT TATCGGG, BAX F: GGGTTGTCGCCCTTTTCTAC, BAX R: GGAGGAAGTCCAATGTCCAG, PUMA F: GAC GACCTCAACGCACAGTA, PUMA R: GTAAGGGCAG GAGTCCCAT, MDM2 F: TGTTGTGAAAGAAGCAGTA GCA, MDM2 R: CCTGATCCAACCAATCACCT, FAS F: TTTCACTTCGGAGGATTGCT, FAS R: TTGATGTCAG TCACTTGGGC, PIRH2 F: CACTGTGAAAACTGTGGA ATTTG, and PIRH2 R: ACACTTGTGTCTTCCTTGA AGATT.

### Luciferase assays

Cells were transfected in 24-well plates (6 × 10^4^ cells/well) using 1 μg of DNA/sample, which included 10 ng of pRL-TK or 1 ng of pRL-SV40 control reporter plasmid (Promega) using Turbofect or Lipofectamine 2000. Samples were processed and analyzed as described [103]. Lysates (12 μl or 30 μl) were also analyzed by Western blot.

### Cell transduction

HEK293T/17 cells were seeded at 6 × 10^6^ cells on 10 cm plates. Cells were transfected with 7.5 pmol pBABE-GFP or pBABE-GFP-HBZ, 20 μg (2.5 pmol) pCL-Ampho and 6.7 μg pHCVG using calcium phosphate. The medium in each plate was replaced with 10 mL of supplemented DMEM 24 hours post-transfection, which was replaced with 6 mL of supplemented RPMI-1640 24 hours later. After an additional 24 hours, the culture medium was passed through a 0.2 μm polysulfone filter and used to transduce 3 × 10^6^ MOLT-4 cells. Three milliliters of supplemented RPMI was added to each 6 mL culture, and Polybrene was then added to a final concentration of 16 ug/mL. Twenty four hours post-transduction, the medium was replaced with 10 mL of supplemented RPMI-1640. Twenty four hours later, cultures were treated with DMSO or etoposide for 5 hours.

### Chromatin immunoprecipitation (ChIP) assays

ChIP assays were performed as previously described [23]. Crosslinked chromatin was extracted in Run-on-Lysis buffer (10 mM Tris-HCl pH 7.5; 10 mM NaCl; 3 mM MgCl_2_; 0.5% NP-40) and then sheared by sonication (Misonix Sonicator 3000) on ice to an average length of 400bp. After pre-clearing with a mix of protein A/G sepharose beads (4°C for 3 hours), the chromatin from an equivalent of 5 × 10^7^ cells was used for immunoprecipitation with antibodies against HBO1 (ab70183, Abcam), which has been characterized [18], and p53 (sc-126, Santa Cruz Biotechnology) or IgG as a control. Immunoprecipitates were eluted in buffer E (25 mM Tris-HCl pH 7.5; 5 mM EDTA; 0.5% SDS) and cross-links reversed at 65°C with proteinase K for 6 hours. Resulting naked DNA was then purified using the QIAquick PCR purification kit (Qiagen) and DNA eluted in 100 μl distilled water.

Quantitative real-time PCR was performed using SYBR Green I. Enrichment for a specific DNA sequence was calculated using the comparative Ct method as previously described [104]. Data are normalized to histone H3 exon 2 background binding and expressed as occupancy value (occupancy). Experiments were performed in triplicate. PCR primer pairs are as follows: BDNF: GTAAAGCCAACCCTGTGTCG, TCCGCTCCAAAATCTGACTC; HOXA9: CGCCAACC AAACACAACAGT, AAGTCGGAAACGACCAACAG; Histone H3: CCAAATGCTGGCATTGTCC, AGTTTT TCCATTTTCATTTGTGTGTG; p21/CDKN1A: GCTG AGCCTCCCTCCATCCCTATGC, TAGAGGTCTCCTGT CTCCTACCATC; p21/CDKN1A p53 RE 5′: AGCAGGC TGTGGCTCTGATT, CAAAATAGCCACCAGCCTCT TCT; p21/CDKN1A p53 RE 3′: CTGTCCTCCCCGAG GTCA, ACATCTCAGGCTGCTCAGAGTCT; p21/CDK N1A TSS: TATATCAGGGCCGCGCTG, GGCTCCA CAAGGAACTGACTTC; p21/CDKN1A coding region: CCAGGAAGGGCGAGGAAA, GGGACCGATCCTAGA CGAACTT and GADD45A: TTCATCTCGCCTGGCTTT TT, AGCAAACAAGGTTTTTGTGGGTT.

### Immunoprecipitation assays

HEK293T/17 (1.5 × 10^6^ cells/10 cm plate) were transfected for 24 hours, and whole cell extracts were prepared as described [51]. Proteins were then immunoprecipitated from 200–400 μg of whole cell extracts as described [93] and analyzed by Western blot. For immunoprecipitation of endogenous HBO1, HBZ-Flag was transfected into H1299 cells for 48 hours, nuclear extracts were then prepared and HBZ was immunoprecipitated with Flag-resin (A2220, Sigma-Aldrich).

### GST pull-down assays

GST, GST-HBZ and GST-HBZ-bZIP were expressed and purified as described [93]. His6-HBO1 was expressed in *E. coli* and purified as described [103]. Purified proteins were dialyzed in HM 0.1 (20 mM HEPES [pH 7.9], 0.1 M KCl, 12.5 mM MgCl2, 1 mM EDTA, 20% [vol/vol] glycerol, 0.025% [vol/vol] Nonidet P40, and 1 mM DTT). Glutathione-agarose beads were equilibrated in 0.5x Superdex buffer (12.5 mM HEPES [pH 7.9], 75 mM KCl, 6.25 mM MgCl2, 5 μM ZnSO4, 20% [vol/vol] glycerol, 0.05% [vol/vol] Nonidet P40, 1 mM EDTA and 1 mM DTT) and incubated with 25 pmol of GST proteins at 4°C for 1 h. Beads were then washed twice with 0.5 × Superdex buffer and combined with 10 pmol of His-HBO1. Binding reactions were mixed at 4°C overnight, and beads were subsequently washed four times with 0.5 × Superdex buffer and resuspended in loading buffer. Eluted proteins were resolved by SDS-PAGE and detected by Western blot.

### *In vitro* HAT assay

HAT assays were performed as described [44] with incubation times and specified protein amounts indicated in the figure legends. Recombinant proteins p300, HBZ, HBZ-AD_1-122_, HBZ-bZIP and histones were described previously [44]. Recombinant protein HBO1 was obtained from SignalChem Pharmaceuticals Inc. (specific activity of 0.4 nmol/min/mg). p53 was expressed in *E. coli*, and the bacterial cell pellet was resuspended in lysis buffer (20 mM Tris [pH 8.0], 0.5 mM EDTA, 0.5 M KCl, 10% [vol/vol] glycerol, 1% [vol/vol] Nonidet P40, 10 mM 2-mercaptoethanol and 0.5 mM PMSF) and sonicated. The cleared lysate was then batch-loaded onto nickel-NTA agarose beads (Qiagen) and eluted with a 10 mM to 0.5 M linear imidazole gradient (supplemented lysis buffer containing 0.1 M KCl). Peak p53 fractions were combined, dialyzed into HM 0.1, batch-loaded onto heparin-agarose beads (Bio-Rad Laboratories), and eluted with a 0.1 to 1 M KCl gradient (supplemented HM). MOZ-HAT was expressed in *E. coli* and purified using Ni-NTA agarose beads (Qiagen) as described [103]. Eluted proteins were dialyzed into HM 0.1.

### Cell cycle and apoptosis analyses

HCT116 p53^+/+^ cells were seeded at 5 × 10^5^ cells in 6 well plates. Unsynchronized cells were treated with drugs or carrier 48 hours after transfection then harvested at indicated time points with phosphate-buffered saline (PBS) and 5 mM EDTA. To synchronize cells, 24 hours after transfection, cultures were switched to serum-free medium for 48 hours. Cells were released from growth-arrest by addition of serum, then treated with drug or carrier before harvesting in PBS/5 mM EDTA at the indicated time points. Cells were washed and resuspended in PBS, fixed in 70% ethanol, stained with 50 mg/mL propidium iodide (Sigma), treated with RNase A (Sigma) and then incubated at 25°C for 30 minutes. Fluorescence was measured by flow cytometry using BD LSR II flow cytometer (BD Biosciences) and data were analyzed with ModFit LT (Verity). For Annexin V/PI staining, 48 hours after transfection, cells were washed and then treated with drug or carrier for 24 hours. Cells were subsequently stained using the Dead Cell Apoptosis Kit with Annexin V FITC and PI for flow cytometry (Life Technologies). Fluorescence was measured by flow cytometry using BD LSR II flow cytometer (BD Biosciences) and data were analyzed with FlowJo (FlowJo LLC).
